# Clinical and Radiological Presentations of Late-Onset Spondyloarthritis

**DOI:** 10.5402/2011/840475

**Published:** 2011-03-20

**Authors:** Ihsane Hmamouchi, Rachid Bahiri, Najia Hajjaj-Hassouni

**Affiliations:** ^1^Laboratory of Information and Research on Bone Diseases (LIRPOS), Department of Rheumatology, Faculty of Medicine and Pharmacy, El Ayachi Hospital, University Hospital of Rabat-Sale, University Mohammed V Souissi, Morocco; ^2^Laboratory of Biostatistical, Clinical and Epidemiological Research (LBRCE), Faculty of Medicine and Pharmacy, University Mohammed V Souissi, Rabat, Morocco

## Abstract

The last few years have witnessed considerable progress in the diagnosis and treatment of spondyloarthritis (SpA). Tools are now available for establishing the diagnosis at an early stage, when appropriate treatment may be able to control the inflammatory process, limit the functional impairments, and improve quality of life. Late-onset SpA after the age of 50 years is uncommon. All the spondyloarthritis subgroups are represented in the elderly. Thus, late onset spondyloarthritis is underdiagnosed in favour of other inflammatory disorders that are more frequently observed in the elderly because the clinical or radiological presentations of late-onset spondyloarthritis are modified in the elderly. They deserve further attention because age population is increasing and new criteria for axial SpA including sacroiliitis detected by MRI may help the clinician with diagnosis. Specific studies evaluating the benefit/risk ratio of TNF*α*-blocking agents in late onset SpA patients are required.

## 1. Introduction

The chronic inflammatory diseases course in the elderly have some specificity regarding their clinical presentation, the presence of comorbidities, and severe illness suggestive of malignancy. This could influence the therapeutic response and the safety of treatment in the elderly. The spondyloarthritis (SpA) involve several disorders sharing common clinical and radiological characteristics with ankylosing spondylitis (AS). Their rheumatic manifestations include spinal symptoms, peripheral arthritis, and enthesopathic lesions. Structural changes usually evolve over years, primarily in the axial skeleton and especially in the sacroiliac joints [1–3]. Ankylosing spondylitis and spondyloarthritis, in their classical onset, are generally observed in young patients; clinical onset after the age of 50 years is uncommon [4]. Previous studies have considered that patients >50 years of age had late-onset disease [4]. However, most epidemiological studies evaluating the safety profile of a treatment define elderly patients as those aged >65 years [5]. 

All the spondyloarthritis subgroups are represented in the elderly: ankylosing spondylitis, reactive arthritis, psoriatic arthritis, articular manifestations of inflammatory bowel diseases, and undifferentiated spondyloarthritis [6–8].

Thus, late-onset spondyloarthritis is underdiagnosed in favour of other inflammatory disorders that are more frequently observed in the elderly because the clinical or radiological presentations of late-onset spondyloarthritis are modified in the elderly [4]. They deserve further attention because age population is increasing and new criteria for axial SpA including sacroiliitis detected by MRI may help the clinician with diagnosis. 

The aim of this paper is to update the clinical and radiological features of late-onset spondyloarthritis.

## 2. The Prevalence of Late-Onset Spondyloarthritis

The prevalence of late-onset spondyloarthritis is not well known. Previous epidemiological series determined a prevalence for late-onset ankylosing spondylitis between 3% and 8% [10]. This prevalence need to be re evaluated because there is new diagnostic criteria for axial SpA recently developed by the Assessment of SpondyloArthritis International Society (ASAS) [9]. Those criteria will facilitate the diagnosis in young patients with inflammatory back pain and also elderly patients. AS or axial SpA can be diagnosed from (i) the presence of sacroiliitis, evident on MRI or radiography, plus at least one SpA feature, or (ii) the presence of HLA-B27 plus at least two SpA features. “Sacroiliitis on imaging” was defined as either active (acute) inflammation on MRI highly suggestive of sacroiliitis associated with SpA or definite radiographic sacroiliitis according to modified New York criteria. “SpA features” included inflammatory back pain, arthritis, enthesitis (heel), uveitis, dactylitis, psoriasis, Crohn's disease/ulcerative colitis, good response to NSAIDs, family history of SpA, HLA-B27, and elevate C-reactive protein (CRP) (Table 1).

## 3. The Clinical Characteristics of Late-Onset Ankylosing Spondylitis or Spondyloarthritis

The clinical spectrum of late-onset spondyloarthritis seems to be as wide as it is in young adults. All the spondyloarthritis subgroups are represented in the elderly.

### 3.1. Ankylosing Spondylitis (AS)

The patients meet the Amor criteria or the European Spondyloarthritis Study Group criteria [11, 12] and they have a predominantly axial disease, with spinal symptoms and sometimes peripheral arthritis. Cervical pain is frequently observed and peripheral arthritis predominates at the lower limbs. Enthesitis (talalgia), dactylitis (sausage toe) or uveitis may occur [13]. Laboratory parameters are usually and markedly elevated. HLA-B27 is positive in 70% of cases [4].

### 3.2. Late-Onset Peripheral Spondyloarthropathy (LOPS)

LOPS was first described by Dubost and Sauvezie [14]. The authors described 10 cases of B27-positive men who developed an oligoarthritis together with a large inflammatory pitting edema of the lower extremities, after the age of 50. All ten patients were male and had moderate involvement of the axial skeleton, oligoarthritis of the lower limbs with pitting oedema in most cases, severe illness with constitutional symptoms, and marked elevation of laboratory parameters of inflammation. All were HLA-B27 positive. Responses to nonspecific nonsteroidal anti-inflammatory drugs were poor and symptoms persisted from 1 to several years. Five patients developed sacroiliitis during followup and four of them met criteria for ankylosing spondylitis. The clinical presentations described in this report were considered to belong to the group of spondyloarthritis.

### 3.3. Undifferentiated Spondyloarthritis (uSPA)

Caplanne et al. [15] compared the clinical presentation of late-onset uSPA with patients with early onset SPA. Eight patients with late-onset SPA were identified after a retrospective chart review of inpatients and outpatients seen over an 8-year period. Late-onset patients had more cervical and dorsal pain, anterior chest wall involvement, peripheral arthritis, aseptic osteitis, and systemic symptoms than patients with early-onset SPA. Two of the eight patients had inflammatory bowel disease.

In 1991, Dubost et al. [16] reviewed the files of male patients admitted in their department over a period of 12 years for rheumatoid factor-negative arthritis beginning after the age of 50. Patients with polymyalgia rheumatica, psoriatic arthritis, or crystal-induced arthritis were excluded. Of the 105 patients, 29 meet American College of Rheumatology criteria for rheumatoid arthritis, 29 met the New York criteria for ankylosing spondylitis, three had reactive arthritis, and 44 had unclassified arthritis. Of these 44, 14 were B27 positive. Most of these latter patients had oligoarthritis together with inflammatory pitting edema, marked constitutional symptoms, and elevated erythrocyte sedimentation rates.

The clinical spectrum of patients with late-onset uSPA was studied by Olivieri et al. [17, 18]. Twenty-three patients (11 men and 12 women; 17 were B27 positive and six were negative) were seen during a 5-year period and followed prospectively. Of these, 12 had three or more manifestations of SpA including peripheral arthritis, peripheral enthesitis, dactylitis, inflammatory spinal pain, buttock pain, chest wall pain, heart involvement, acute anterior uveitis, and sacroiliitis. Seven patients showed two manifestations and four showed only one. Only 10 of the 23 patients had peripheral arthritis, three of whom had ankle or tarsus involvement together with the large inflammatory pitting edema described by Dubost and Sauvezie. Of the two patients with only one manifestation, two had peripheral enthesitis, and two had acute anterior uveitis. Of the 23 patients, only 15 met the ESSG or the Amor criteria for SpA. The clinical spectrum is found as wide as in children and middle age adults and includes patients with pitting edema. No association with inflammatory bowel disease or psoriasis was present. 

In 2007, the same authors described seven cases of uSPA in patients presenting polymyalgia rheumatica (PMR) features [19]. All patients with late-onset uSPA meeting criteria for PMR at the onset of their disease were seen. All patients had manifestations of SPA at the beginning of the disease and two developed these in the following 6 months. All seven met the Amor and/or the ESSG criteria for classifying and diagnosing SPA. The conclusion was that late-onset uSPA may have PMR-like features at the beginning of the disease and that the diagnosis is not difficult if the entire clinical spectrum of SpA is considered.

### 3.4. Others Forms of Late-Onset SpA

Previous study estimated the frequency of late-onset reactive arthritis at 13% [10]. Four reactive arthritis patients among 105 inflammatory arthritis patients >50 years of age were reported by Dubost et al. [20].

In the elderly, psoriatic arthritis is considered to be more severe and to have a less favorable outcome than young-onset psoriatic arthritis, suggesting that there is more frequent use of aggressive or innovative treatments such as anti-TNF*α* in older patients [21].

Punzi et al. [21] have prospectively evaluated the presenting manifestations and the 2-year outcome of 60 consecutive patients with psoriatic arthritis, 16 of whom had elderly onset and 50 of whom had younger onset. The elderly group had a significantly higher number of active joints, foot erosions, and levels of serum C-reactive protein and synovial interleukin-1 and interleukin-6 than younger patients. After 2 years the progression rate of the joint damage and the C-reactive protein level were higher in elderly patients than in younger ones. 

In Cantini et al. inflammatory pitting edema on the dorsum of hands or feet was found in 39 of 183 patients (21%) with psoriatic arthritis and in 18 of 366 controls (4.9%) [22]. The lower limbs were more frequently involved than the upper ones asymmetrically.

## 4. The Imaging Characteristics of Late-Onset Spondyloarthritis

### 4.1. Contribution of Standard Radiographs

Standard radiographs to look for sacroiliitis or syndesmophytes contribute little to the diagnosis of late-onset SpA, as changes induced by age (osteoporosis, osteoarthritis, and discarthrosis make it difficult to interpret X-Rays [4].

### 4.2. Contribution of Magnetic Resonance Imaging

The introduction of MRI as a diagnostic investigation for SpA is a major advance. MRI is a very helpful imaging method for the early diagnosis of AS, that is, at a pre- or nonradiographic stage [24]. MRI is a useful diagnostic tool because it has good specificity (88% to 98.5%). However, MRI may have limited sensitivity for detecting low-grade inflammation (32%–50%) [25–30].

There has been no specific study of MRI assessment of the sacroiliac joints or the spine in patients with late-onset SpA, but it is probable that the MRI findings will certainly be the same as in the young population. Indeed, similar MRI changes were seen in 20% of healthy individuals without inflammatory disease, and also in patients with discarthrosis or malignant bone disease (metastasis). Combining MRI of the sacroiliac joints and spine provides more information than MRI of either site alone [5]. 

No studies have evaluated the diagnostic performance of MRI for diagnosing SpA in patients who are HLA B27-negative and who fail to meet criteria for inflammatory low back pain [31]. Most of the studies in which MRI contributed to the diagnosis of recent-onset SpA were conducted in HLA B27-positive patients who had chronic inflammatory low back pain [27, 32].

### 4.3. Contribution of Ultrasonography

In patients with LOPS, ultrasound assessment of joint structures may be useful for evaluating synovial and tenosynovial modifications [5]. Ultrasonography is a simple and inexpensive investigation that is more sensitive than the physical examination for diagnosing enthesitis related to SpA. Two recent studies have provided data on the performance of color Doppler ultrasonography for assisting in the diagnosis of SPA by evaluating inflammation of the sacroiliac joints and spine [33, 34]. No study has been done to evaluate the accuracy of this technique in that range of patients.

## 5. Differential Diagnosis

The diagnosis of late-onset axial SpA may be easier than LOPS but care must be taken not to mistake spinal findings. In fact, the recognition of sacroiliitis by standard radiographs is more problematic because of changes induced by age (osteoporosis, osteoarthritis, and discarthrosis). DISH and SpA have in common the involvement of axial skeleton and extraspinal entheses but their radiological features are different [35, 36]. MRI is a very helpful imaging method for the diagnosis of axial SpA. Diffuse idiopathic skeletal hyperostosis (DISH) [37].

In contrast, a LOPS may be more difficult to diagnosis with other inflammatory diseases in which remitting distal extremity swelling with pitting edema has been observed include chondrocalcinosis, amyloid arthropathy, systemic lupus erythematosus, mixed connective disease, Sjögren syndrome, systemic sclerosis, dermatomyositis, and polyarteritis nodosa [38–44].

RS3PE (remitting seronegative symmetrical synovitis with pitting edema) syndrome [45, 46] is characterized by an acute onset of bilateral symmetric synovitis involving predominantly the wrist, the carpus, the small hand joints, and the flexor digitorum sheaths associated with a marked dorsal swelling of the hands with pitting edema (“boxing glove hand”). Patients are persistently seronegative for rheumatoid factors and show elevated acute-phase reactants. The disease is sensitive to small doses of steroids and remains in remission after such therapy.

Since late-onset ankylosing spondylitis or spondyloarthropathy may give rise to polymyalgia rheumatica-like manifestations, this differential diagnosis should be considered [47]. The first point in the differential diagnosis between late-onset uSpA and PMR is the presence of inflammatory swelling with pitting edema due to tenosynovitis of the extensor tendons of hand or foot in both conditions [48]. The second point is the possibility that late-onset uSpA may begin with pain and stillness in the shoulders and hip girdles mimicking PMR [19].

## 6. Anti-TNF*α* Agents in Late-Onset Spondyloarthritis

In clinical trials evaluating the efficacy of anti-TNF*α* agents in AS, patients >65 years of age were generally excluded. Thus, data on the efficacy and safety of anti-TNF*α* agents in late-onset SpA are lacking. Indirect experience of the use of TNF*α* blockers in the elderly derives from studies in RA, although RA and AS patients are not comparable [5]. 

Based on the available literature, some recommendations for the use of anti-TNF*α* agents in elderly RA patients have been proposed [49, 50]. These recommendations, which are also applicable to patients with late-onset SpA, are careful selection of the patient before initiating the TNF*α*-blocking agent, evaluation of comorbidities, and estimation of the risk for severe and opportunistic infections [5].

## 7. Keys Points

1. The spondyloarthritis are most typically seen in younger patients. However, late-onset SpA after the age of 50 years is uncommon.
2. The clinical spectrum of late-onset AS and SpA seems to be as wide as it is in young adults.
3. Two main clinical presentations.
  - The patients may have a predominantly axial disease, with spinal symptoms and sometimes peripheral arthritis. Cervical pain is frequently observed and peripheral arthritis predominates at the lower limbs. Enthesitis (talalgia), dactylitis (sausage toe) or uveitis may occur. Laboratory parameters are usually and markedly elevated. HLA-B27 is positive in 70% of cases.
  - The patient may present with late-onset peripheral spondyloarthritis (LOPS) with distal inflammatory swelling with pitting edema on the dorsum of feet or hands together with peripheral arthritis and peripheral enthesitis.
4. Some patients show only one manifestation of the B27-associated disease process for years and need to be evaluated by the new diagnostic criteria for axial SpA (Table 1).
5. If the clinical features do not immediately establish the diagnosis, an anterior posterior radiograph of the pelvis is useful to look for sacroiliitis. When this investigation fails to show sacroiliitis, the authors recommend MRI of the sacroiliac joints and thoracolumbar spine to look for inflammation, in keeping with recent recommendations (Table 2).
6. Doppler ultrasonography can also contribute to the diagnosis of enthesitis and peripheral synovitis.
7. LOPS may be difficult in diagnosis especially with RS3PE syndrome and polymyalgia rheumatica.
8. Specific studies evaluating the benefit/risk ratio of TNF*α*-blocking agents in late-onset SpA patients are required.

## Figures and Tables

**Table 1:** ASAS criteria for axial spondyloarthropathy [9].

Back pain ≥3 months and age <45 years ET		
Sacroiliitis by MRI or radiography* + 1 other feature	OR	HLA B27 + 2 other features
*Features of axial SpA*		
Inflammatory back pain	4 of the 5 following characteristics	
	Age <40 years	
	Insidious onset	
	Improvement with exercise	
	No improvement with rest	
	Pain at night	
Arthritis	Past or present active synovitis diagnosed by a physician	
Enthesitis	Pain spontaneously or upon palpation of the Achilles tendon insertion site or plantar fascia	
Uveitis	Past or present anterior uveitis diagnosed by a physician	
Dactylitis	Past or present active dactylitis diagnosed by a physician	
Psoriasis	Past or present active psoriasis diagnosed by a physician	
Crohn's disease/ulcerative colitis	Past or present, diagnosed by a physician	
Good response to NSAIDs	24–48 h after the initiation of full-dose NSAID therapy, the pain is gone or much better	
Family history for SpA	First- or second-degree relative with any of the following: SpA, psoriasis, acute uveitis, reactive arthritis, chronic inflammatory bowel disease	
HLA-B27	Presence of B27	
Elevated CRP	CRP above the upper limit of the normal range, in the absence of another cause of CRP elevation	

*Sacroiliitis (X-rays or MRI): Definite radiographic sacroiliitis (grade 2 bilaterally or grade 3-4 unilaterally; according to modified New York criteria 1984) Or—active (acute) inflammation of sacroiliac joints on MRI, highly suggestive of sacroiliitis associated with spondyloarthritis.

**Table 2:** Recommendations for imaging studies in patients with spondyloarthropathy—2006 Meeting of Rheumatology Experts [23].

	Level of evidence	Grade of recommendation	Agreement among experts (%)
The diagnosis of ankylosing spondylitis requires standard radiographs of the pelvis (anteroposterior view) and lumbar spine (anteroposterior and lateral views including the thoracolumbar junction).	2b	D	92.8
When standard radiographs conclusively demonstrate bilateral sacroiliitis, further imaging studies are not necessary for establishing the diagnosis of ankylosing spondylitis.	—	D	90.1
When radiographs are normal or doubtful in a patient with a clinical suspicion of ankylosing spondylitis, diagnostic MRI of the sacroiliac joints is recommended.	2a	B	98.7
MRI of the spine can contribute to the diagnosis of ankylosing spondylitis in patients who have inflammatory back pain with nonsuggestive radiographs of the pelvis and spine.	3	C	98.6
To evaluate entheseal involvement in patients with a clinical suspicion of ankylosing spondylitis, radiographs may be useful and, if needed, Doppler ultrasonography or MRI may deserve to be performed, or radionuclide scanning when multiple entheses are involved.	2b/3	D	81.7
Given the current state of knowledge, imaging methods other than standard radiography are not useful to predict the functional or structural outcome of ankylosing spondylitis.	2b	D	94.4
Given the current state of knowledge, imaging is not appropriate for the routine followup of patients with ankylosing spondylitis. Instead, additional imaging should be performed as dictated by the clinical course.	2a	C	95.1
Given the current state of knowledge, imaging is not recommended for evaluating treatment responses in patients with ankylosing spondylitis.	1b/2b	C	97.1
